# Questions Submitted for Discussion by Local Societies—Formulated by the Committee Appointed by the American Dental Association

**Published:** 1897-05

**Authors:** 


### QUESTIONS.

### Questions Submitted for Discussion by Local Societies—Formulated by the Committee Appointed by the American Dental Association.

1. Pyorrhea alveolaris; what is it, and how many varieties are there? Are all local in origin or constitutional, or both? What is the treatment, local or constitutional, or both? What may be regarded as a cure? Is the disease likely to recur?

2. *a*—What is the cause of dental caries? *b*—Why is caries so much more active in some mouths than in others? *c*—What changes take place where caries ceases its activity in mouths heretofore predisposed? *d*—Are there recognizable signs by which we may know whether or not caries will cease with advancing age?

3. To what extent are we justified in giving our patients systematic treatment?

4. To what extent, and when are we justified in using cataphoresis? Is there danger of injuring the dental pulp or other tissues by its use?

5. What can we do to increase the attendance at our dental societies?

6. In view of the recent investigations has amalgam been a blessing or a curse to humanity?

7. Are there any proofs that the mercury in amalgam fillings is injurious to the health of the patient?

8. What are the best materials for filling teeth, and the prospective durability of fillings in different cases?

9. What are the best methods of bleaching teeth?

L. P. BETHEL,<br>A. W. HARLAN, } *Committee.*<br>J. N. CROUSE.

